# An Economic Comparison of Influenza Vaccines Recommended for Use in Eligible Adults under 65 Years in the United Kingdom

**DOI:** 10.3390/vaccines10040599

**Published:** 2022-04-13

**Authors:** Michael Maschio, Michele A. Kohli, Mansoor Ashraf, Michael F. Drummond, Milton C. Weinstein, Joaquin F. Mould-Quevedo

**Affiliations:** 1 Quadrant Health Economics Inc., 92 Cottonwood Crescent, Cambridge, ON N1T 2J1, Canada; michael.maschio@quadranthe.com; 2 Seqirus UK, Point, Level 3, 8AA, 29 Market St., Maidenhead SL6 8AD, UK; mansoor.ashraf@seqirus.com; 3 Centre for Health Economics, University of York, Heslington, York YO10 5DD, UK; mike.drummond@york.ac.uk; 4 Harvard T.H. Chan School of Public Health, 718 Huntington Avenue, Boston, MA 02115, USA; mcw@hsph.harvard.edu; 5 Seqirus USA Inc., 25 Deforest Avenue, Summit, NJ 07901, USA; joaquin.mould-quevedo@seqirus.com

**Keywords:** influenza vaccine, cost-effectiveness, economic modeling

## Abstract

**Background**: In the United Kingdom (UK), a cell-based quadrivalent influenza vaccine (QIVc) and a recombinant vaccine (QIVr) are recommended for eligible adults under 65 years. The objective of this analysis was to determine the potential cost-effectiveness of QIVc compared to QIVr for this age group using a range of assumptions about relative vaccine effectiveness (rVE). **Methods:** A dynamic transmission model, calibrated to match infection data from the UK, was used to estimate the clinical and economic impact of vaccination across 10 influenza seasons. The list price was £12.50 for QIVc and £22.00 for QIVr. The base case effectiveness of QIVc was 63.9%. As there are no data comparing the vaccines in the 18 to 64-year-old age group, rVE was varied. **Results:** For the base case, the rVE of QIVr compared with QIVc must be at least 25% in order for the cost per quality-adjusted life-year gained to be £20,000 or lower. Sensitivity analysis demonstrated that the rVE required for QIVr to be cost-effective was most dependent on the absolute effectiveness of QIVc. **Conclusion:** At list prices, our analysis predicts that the rVE for QIVr must be at least 25% compared to QIVc in order to be considered cost-effective.

## 1. Introduction

Infectious disease experts are increasingly concerned about the potential egg adaptation that may occur in standard influenza vaccines that are cultured in hens’ eggs [1]. Some strains of influenza mutate so that they grow better within the egg, reducing their match to circulating influenza strains. The greatest concern is with AH3N2, which may cause more severe morbidity and mortality than other influenza subtypes. In the United Kingdom (UK), the Joint Committee on Vaccination and Immunisation (JCVI) noted that the egg adaptation may impact vaccine effectiveness against AH3N2 as often as every other year [2]. Given these concerns, they have started to recommend the use of vaccines that are not cultured in hens’ eggs.

In the UK, there are currently two vaccines that are developed with a novel manufacturing process in order to avoid this potential egg adaptation. A quadrivalent influenza vaccine which is cultured in mammalian cell lines (QIVc) (Flucelvax Tetra^®^, Seqirus, Maidenhead, UK) [3] has been available for several influenza seasons and may be used in individuals aged 2 years and older. More recently, a quadrivalent recombinant vaccine (QIVr) has also been authorised for use in the UK in those 18 years old and above (Supemtek^®^, Sanofi Pasteur, Reading, UK) [4]. To create this recombinant influenza vaccine, a gene for the influenza surface protein called hemagglutinin (HA) is combined with a baculovirus genome and expressed in insect cell lines. The JCVI has now advised the use of both vaccines in eligible individuals aged 18 to 64 years for the 2021/22 and 2022/23 influenza seasons [5,6,7,8]. 

As QIVc has been used in the UK in the 18 to 64 years age group since the 2019/20 season, [5,6,7,8] data on the effectiveness of this vaccine are available. During the 2019/20 season, Public Health England (PHE) estimated the effectiveness of QIVc against all influenza types to be 63.9% (95% confidence interval of 26.9–82.2%) in a test-negative case control study [9]. The effectiveness of the quadrivalent standard egg-based influenza vaccines (QIVe) in the same age group was only 38.9% (95% confidence interval of −4.5% to 64.3%). The data for QIVr for this age group come from a randomized controlled trial (RCT) that was conducted during the 2014/2015 influenza season in adults ages 50 years and above. For this trial, those receiving QIVr had a statistically lower rate of influenza infections than those receiving QIVe. The relative vaccine effectiveness (rVE) of QIVr vs. QIVe, defined as one minus the ratio of the incidence rates of the two vaccines, was 30% (95% confidence interval: 10–47%) [10]. There was one RCT among healthcare personnel aged 18 to 64 years that compared the antibody responses of QIVc and QIVr to QIVe, but infection outcomes were not tracked [11]. There are no other head-to-head studies of QIVr and QIVc in this age group [12].

Given that the evidence suggests that both QIVc and QIVr may be more effective than QIVe in those 18 to 64 years old, the JCVI advises use of either of these vaccines for eligible individuals in this age group. There is, however, a price difference between the two vaccines which may impact their relative value. The objective of this analysis was to determine the potential cost-effectiveness of vaccination of adults aged 18 to 64 years with QIVc compared to QIVr in the UK using a range of assumptions about the rVE of the vaccines.

## 2. Methods

The main target population for this economic analysis consists of individuals aged 18 to 64 years in the UK eligible for vaccination in the 2021/22 influenza recommendations. In the past, the UK has funded influenza vaccination in this age group only for those considered to be at higher risk of experiencing a more severe case of influenza. In the 2020/21 influenza season, however, the UK implemented a temporary recommendation to expand vaccination to all individuals aged 50 to 64 years in order to reduce the burden of hospitalisation during a season in which influenza and coronavirus disease 2019 (COVID-19) were co-circulating [13]. This measure has also been adopted for the 2021/22 season [14]. In a previous analysis, we had estimated that this measure would be cost-effective if adopted on a permanent basis [15]. Given this distinction, a sub-group analysis was therefore conducted to examine the results for the 50 to 64 year age group alone.

For this analysis, we predicted the number of influenza infections using a compartmental transmission model with a susceptible–exposed–infectious–recovered (SEIR) structure. The model structure and the calibration of the inputs for the transmission model have been described in detail previously [15,16]. Briefly, as is standard for SEIR models, individuals move from susceptible to exposed compartments according to the force of infection, which is a function of the rates of effective contacts between susceptible and unsusceptible individuals in the population. Effective contact is a function of the age-specific contact matrix and the transmissibility of the virus per contact. Time spent in the exposed and infected compartments is a function of the duration of latent and infectious periods associated with influenza. Each of the compartments are stratified into 15 age groups and by risk of complication from infection (low or at-risk). Each influenza season is treated independently, as are infections with influenza A and B. The model inputs for transmissibility, susceptibility and percent of infected cases with clinical symptoms were derived through a calibration process which produced an average influenza A season and an average season in which influenza A and B are co-circulating using data and results from previous modeling exercises from the UK. 

For the current analysis, we compared the use of QIVc and QIVr in both the full 18 to 64-year-old target population and the 50 to 64-year-old sub-population in the UK. Given its structure, which allows for transmission across age groups, the model included the entire UK population and therefore required that the recommended vaccination policy be modelled for all age groups. As in the previously published analysis [15], QIVe was used for those 6 to 23 months, quadrivalent live-attenuated influenza vaccine (QLAIV) for those aged 2 to 17 years and adjuvanted quadrivalent for those 65 years and older. QIVc was considered to be the default vaccine for those aged 18 to 64 years old as it has been used for several seasons. QIVr was substituted for QIVc in the 18 to 64-year-old age group or the 50 to 64-year-old age group as applicable to conduct our current comparisons.

The perspective used for the study was that of the National Health System (NHS) and Personal Social Services as recommended by the National Institute for Health and Care Excellence (NICE) [17]. A discount rate of 3.5% for both costs and outcomes was in line with NICE recommendations [17].

The costs and consequences of each infection were estimated using a decision tree as described in our previous analysis [15]. Briefly, the rate of hospitalisation and the associated risk of mortality while in hospital were estimated using data from the UK [18]. In scenario analyses, we doubled and halved the rate of hospitalisation. Resources for the outpatient treatment of influenza were based upon previous UK analyses [19,20,21]. The impact of influenza on quality of life was captured as a quality-adjusted life-year (QALY) decrement derived from past studies [22,23,24]. The impact of a death due to influenza was captured as discounted QALYs lost, calculated using expected survival [25] and mean age-specific utility values [26]. All model inputs are the same as those previously described [15], except for the vaccine unit prices for QIVc and QIVr, vaccine coverage and vaccine effectiveness, as described below.

For the base case analysis, the unit costs of vaccines were based on the current list prices: £12.50 for QIVc and £22.00 for QIVr [3,4]. We conducted scenario analyses in which the price of QIVr was reduced to £20.00 and £18.00 to determine the impact of a potential price change on the unit cost of the vaccine provided by the manufacturer. We did not include vaccine administration costs, as they were expected to be same regardless of the vaccine given. Vaccine coverage inputs were updated for those ages 50 years and above to reflect the observed vaccine uptake rates from the 2020/21 season in the UK, during which vaccination for all individuals aged 50 to 64 years was funded. All other coverage inputs [27] and the percent of individuals considered to be at-risk due to complications [22,28,29] in each age group were not changed (Table 1). 

Consistent with our previously published analysis [15], we set the effectiveness of QIVc to 63.9% based on the data from PHE [9]. We conducted scenario analyses using the 95% confidence intervals of QIVc effectiveness from that study (26.9% and 82.2%). Additionally, in scenario analyses we doubled and halved the hospitalization rates, as we had identified severity of influenza as one of the important drivers of cost-effectiveness in our previous publication [15]. For all analyses, the rVE of QIVr compared to QIVc was varied across a wide range of values to determine the value at which QIVr would be cost-effective compared to QIVc given its higher price. We used the willingness-to-pay threshold of £20,000 per QALY gained, as the JCVI considers interventions with an incremental cost-effectiveness ratio (ICER) below this to be cost-effective [30]. For our base case reference scenario, we also tested what rVE would be required to achieve cost-savings for QIVr (willingness-to-pay threshold of £0 per QALY gained).

As in the previous publication [15], we modelled 10 influenza seasons using data available for the 2010/11 to 2019/20 seasons from the Public Health England (PHE) surveillance reports [9,31,32,33,34,35,36,37,38,39]. All outcomes are presented as averages per season over the 10 years.

## 3. Results

**Figure 1:**
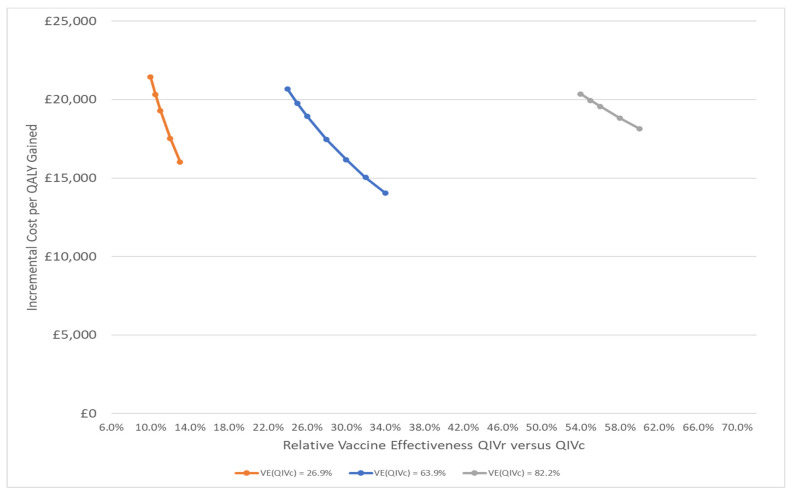
Impact of relative vaccine effectiveness on the incremental cost per quality-adjusted life-year (QALY) gained of QIVr compared to QIVc for different levels of absolute effectiveness (VE) of QIVc.

In the base case analysis, with a QIVc price of £12.50 and a QIVr price of £22.00, the rVE of QIVr compared with QIVc must be at least 25% (implying an absolute effectiveness of 72.9% for QIVr) in order for the cost per QALY gained to be £20,000 or lower. We attempted to find an rVE where QIVr is cost saving compared to QIVc but we found that was not possible: for all rVEs, further reductions in influenza costs were not sufficient to offset the higher unit cost of QIVr. The impact of varying the absolute effectiveness of QIVc, the rate of hospitalisation and the unit price of QIVr is shown graphically in Figure 1. Varying vaccine effectiveness has the highest impact on the results. If the absolute effectiveness of QIVc is reduced to 26.9%, then a lower rVE of QIVr versus QIVc of 11% (absolute effectiveness of 34.9% for QIVr) is required to achieve the willingness-to-pay threshold. Conversely, if the absolute effectiveness of QIVc is increased to 82.2%, then a greater rVE of QIVr versus QIVc of 55% is required (absolute effectiveness of QIVr of 92.0%). When we increased the severity of the impact of influenza by doubling hospitalisations, QIVr became cost-effective with a lower rVE of 15% (absolute effectiveness of 69.3%). However, in milder years, represented by a 50% reduction in the rate of hospitalisation, QIVr must achieve an rVE of 36% (absolute effectiveness of 76.9%) in order to be cost-effective. If the cost difference between the two vaccines decreases due to price changes, then the effectiveness of QIVr does not need to be as high to be cost-effective. Our model predicts that with a unit cost of £20.00 for QIVr, for example, the rVE of QIVr compared to QIVc required to achieve the threshold of £20,000 declines to 19% (absolute effectiveness of 70.8%). For a unit price of £18.00, the rVE required is 14.5% (absolute effectiveness of 69.1%).

**Figure 2:**
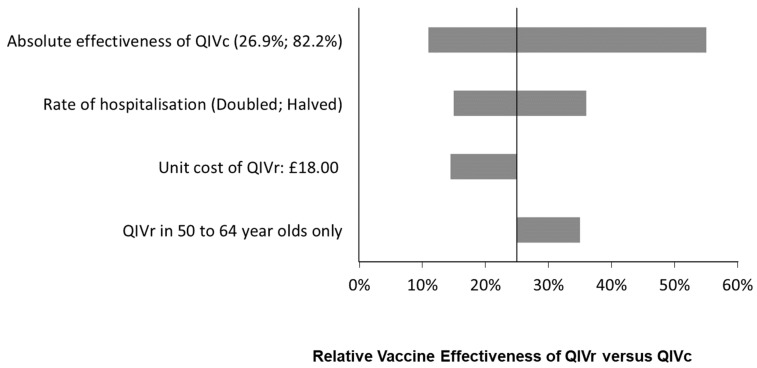
Tornado diagram: impact of varying key inputs on the minimum relative vaccine effectiveness of QIVr compared to QIVc required to achieve a cost per QALY gained of £20,000 or lower.

In a sub-group analysis examining only 50 to 64-year-olds, a higher relative effectiveness was required when offering QIVr to the 50 to 64-year-olds only compared to implementation in all 18 to 64-year-olds. As shown in Figure 2, with a £20,000 willingness-to-pay threshold, the rVE of QIVr vs. QIVc must be at least 35% (absolute effectiveness of 76.5%).

## 4. Discussion

In this analysis, we looked at the impact of varying the relative vaccine effectiveness of QIVr compared to QIVc on the incremental cost per QALY gained. We determined that in order to achieve an ICER of £20,000 per QALY gained, the rVE needed to be at least 25% under base case conditions. As in previous analyses that we have conducted [15,16], the absolute effectiveness of the less expensive vaccine is an important driver of the value of the more effective and expensive vaccine. In years where QIVc has lower efficacy, the value of a more effective vaccine is greater: smaller values of the rVE allow the vaccine to achieve the required level of cost-effectiveness. Similarly, when influenza causes more severe outcomes, smaller values of rVE are more valuable and prevent more morbidity and health care costs. In less severe influenza years, or if QIVc is more effective in preventing disease, a more expensive vaccine such as QIVr needs to be more effective when it comes to reducing morbidity, mortality and costs in order to justify an increased unit cost in the cost per QALY framework.

There is no head-to-head study comparing the impact of QIVc and QIVr on infection in the 18 to 64-year-old age group. For the base case analysis, QIVr absolute effectiveness would need to be 73% in order to achieve an rVE of 25% compared to QIVc. In the future, data from sources such as the ongoing PAIVED trial, a pragmatic assessment over three influenza seasons amongst US Department of Defense beneficiaries aged 18 and over, should provide further information about the rVE of the two vaccines.

As with any decision analysis, there are several limitations associated with our analysis comparing QIVc and QIVr. We have discussed the limitations associated with the model structure and inputs in our previous analyses [15,16]. The main limitation is that while we have used type distribution data from 10 different years, we have calibrated the model to only two general kinds of seasons: one that was primarily type A and one that was a mix of types A and B. Our model may therefore underestimate the variability associated with influenza from season to season. Furthermore, we do not know how severe influenza seasons will become in future seasons after the COVID-19 pandemic has ended. We have conducted sensitivity analyses by widely varying the rate of hospitalisation in order to assess the impact of this limitation and to speculate on what may happen if influenza becomes more or less severe after the COVID-19 pandemic. As we assumed that all deaths occur post hospitalisation, varying the rate of hospitalisations also varies the number of deaths predicted by our model [15,16].

## 5. Conclusions

The JCVI advises the use of either QIVc and QIVr for eligible individuals aged 18 to 64 years for the 2021/22 and 2022/23 seasons in the UK. Data comparing the relative vaccine effectiveness of QIVr to QIVc are sparse. At the current list prices of these vaccines, our analysis predicts that the rVE of QIVr compared to QIVc must be at least 25% in order for QIVr to be considered cost-effective. 

## Figures and Tables

**Table 1:** Vaccine coverage inputs.

Age Group	Percent of Population at High Risk of Complication if Infected	Vaccine Coverage (%)	
		Low Risk	High Risk
6–23 months	4.90%	0.10%	3.10%
2–6 years ^1^	7.30%	28.10%	48.60%
7–17 years ^1^	9.60%	27.60%	48.60%
18–49 years	9.10%	0.00%	48.60%
50–59 years	18.30%	34.0%	52.00%
60–64 years	18.30%	34.0%	52.00%
65–74 years	45.00%	81.00%	81.00%
75 years and above	45.00%	81.00%	81.00%

^1^: The influenza vaccine programme for children has been expanding and therefore these estimates from Thorrington et al., 2019 [27] now likely underestimate current coverage levels.

## Data Availability

Not applicable.
